# Long-term follow-up demonstrates change in conformation shape of the focal choroidal excavation lesions

**DOI:** 10.1186/s12886-024-03415-8

**Published:** 2024-04-02

**Authors:** Zuzana Sulavikova, Zuzana Sustykevicova, Marek Kacerik, Igor Kozak

**Affiliations:** 1 Department of Ophthalmology, Faculty hospital Trencin, Legionarska 28, 91101 Trencin, Slovakia; 2 Moorfields Eye Hospitals UAE, Abu Dhabi, United Arab Emirates

**Keywords:** Conformity change, Focal choroidal excavation, Imaging, Pachychoroid

## Abstract

**Purpose:**

This study aims to present long-term observation of 5 eyes with focal choroidal excavation (FCE), focusing on morphological changes in conformity of the lesion.

**Methods:**

A retrospective case series was conducted, including 5 eyes of 5 patients with FCE. The study utilized multimodal imaging including color fundus photography, optical coherence tomography (OCT), enhanced depth imaging OCT (EDI-OCT), fundus fluorescein angiography (FFA), fundus autofluorescence (FAF), red free imaging, and OCT angiography.

**Results:**

The mean age at diagnosis was 51 ± 10.65 years, with a mean follow-up period 37 ± 13.59 months. All cases were unilateral, with 1 presenting FCE as an isolated lesion, and one patient exhibiting 2 FCEs in one eye. The mean choroidal thickness measured by EDI-OCT was 268.2 ± 63.39 μm in the affected eye. One patient displayed choroidal thickening and pachyvessels. Of the 5 eyes, one had conforming and 4 non-conforming FCE. We observed a conversion in conformity in all patients, with 4 cases transitioning from non-conforming FCE to conforming type (3 spontaneously, 1 treatment-induced). In conforming FCE, a hyporeflective space appeared twice between neuroretina and retinal pigment epithelium with spontaneous regression.

**Conclusion:**

We observed change in shape from the conforming to non-conforming FCE and vice versa in all patients. We consider this small change in the hyporeflective space as non-pathologic and clinically insignificant.

## Introduction

Focal choroidal excavation (FCE) was initially reported by Jampol in 2006 in a 62-year-old myopic patient without visual symptoms [1]. Margolis first described the bilateral occurrence and defined the term “focal choroidal excavation” [2]. This condition manifests as a concavity of the choroid, without scleral ectasia or staphyloma, often identified through a characteristic appearance on optical coherence tomography (OCT) [1–5]. Based on the OCT characteristics, FCE is classified into two types: conforming and non-conforming. Conforming FCE exhibits no separation between photoreceptor outer segments and the retinal pigment epithelium (RPE). In contrast, non-conforming FCE features detached photoreceptors from the underlying RPE, creating a hyporeflective space [2–7]. Shinojima et al. further categorized FCE into three morphological patterns based on OCT appearance: cone-shaped, bowl-shaped, or mixed-type [7].

Despite its likely underestimation due to the absence of visual symptoms and atypical fundus presentation [6], the precise incidence and prevalence of FCE in the population remain unknown. Extrafoveal FCE typically lacks clinical symptoms, while subfoveolar FCE may cause a slight decrease in visual acuity, microscotomas, and metamorphopsia [3, 4].

The etiology and pathogenesis of FCE remain unclear. While some cases may be congenital embryological chorioretinal disorder, the majority are acquired, often forming at sites of other chorioretinal diseases [3, 4, 8–12]. The low prevalence of FCE among younger patients strongly supports the perspective that FCE is acquired lesion [13]. Gan‘s study indicates that 54.79% FCEs appear within the lesions of the underlying disease such as choroidal osteoma, punctate inner choroidopathy and central serous chorioretinopathy (CSCR), and approximately 28.77% are situated at the edges of such lesions [14].

FCE belongs to the pachychoroid spectrum diseases, characterized by increased choroidal thickness (above 300 μm) and dilatation of the Haller’s layer vessels (referred to as pachyvessels), which compress the overlying choriocapillaris and Sattler’s layer. The choroidal thickening can be focal or diffuse, foveal or extrafoveal [15–18].

Multimodal imaging is crucial for FCE detection and monitoring due to non-specific findings in fundoscopy often presenting as atypical pigmentary or yellow changes [6]. Spectral domain OCT provides detailed visualization of the retinal structure and is used to identify associated maculopathies and define FCE type [19]. OCT angiography offers non-invasive imaging of the retinal and choroidal vessels, while enhanced depth imaging OCT (EDI-OCT) help us visualize deeper structures and evaluate the choroid both qualitatively and quantitatively. These OCT modalities demonstrate focal or diffuse choroidal thickening, pachyvessels and thinning of the inner choroid layers (choriocapillaris and Sattler’s layer) [20–22]. Fundus autofluorescence (FAF) reveals varying degrees of hyper or hypoautofluorescence, corresponding to RPE damage. Both OCT angiography and fundus fluorescein angiography (FFA) are helpful in diagnosing associated chorioretinal neovascularization (CNV) [19].

The long-term stability or conversion of morphological shapes in FCE remains unclear. This report aims to provide a comprehensive, long-term observation of FCE, emphasizing changes in morphological shape and conformity of the lesion.

## Methods

This retrospective case series included 5 eyes of 5 patients with a FCE. Diagnosis was confirmed by multimodal imaging, and follow-up was conducted at Ophthalmology clinic in the Faculty hospital Trencin, Slovakia from August 2018 to June 2023. During scheduled visits, comprehensive ophthalmic exatimantions was conducted for all patients. This included best-corrected visual acuity (BCVA), slit lamp biomicroscopy, fundus examination with artificial mydriasis and non-contact intraocular pressure measurement. Spectral domain OCT (Spectralis Heidelberg, Germany), OCT angiography and EDI-OCT (XR Avanti AngioVue OCTA, Optovue, Fremont, USA) were performed using 6 × 6 mm macula images with automatic segmentation mode. Excluded from the analysis were images of low-quality, artefacts, those with uncorrected segmentation, or blurred images. Choroidal thickness was measured manually by EDI-OCT under the fovea and in the region of maximum choroidal thickening in the affected eye. OCT angiography was used to identify chorioretinal vascular abnormalities and CNV. Fundus photography, FAF, FFA and red free imaging were performed by digital retinal camera (Canon CX-1, Tokyo, Japan). Patients underwent regular follow-up visits at intervals of 3–6 months, depending on the findings, and performed self-monitoring using the Amsler test. This study adhered to the principles of the Declaration of Helsinki.

## Results

Five Caucasian patients (2 females and 3 males) were included in this study (Table 1). The mean age (± standard deviation) of the group at the time of diagnosis was 51 ± 10.65 years (range: 37–63), with an average follow-up period of 37 ± 13.59 months. In all eyes FCE was observed unilaterally, with 4 cases presenting as an isolated lesion and in 1 patient having 2 FCEs in one eye. Visual complaints related to FCE were reported by 2 out of 5 patients, while FCE was typically discovered incidentally during routine eye examination. The mean refractive error was − 0.4 ± 4.03 diopters (ranging from − 5.0 to + 4.0), with 2 myopic eyes, 1 emmetropic and 2 hyperopic. In 4 cases, fellow eyes did not show any macular pathology, while in 1 case pachychoroid pigment epitelopathy was detected. The mean choroidal thickness, measured by EDI-OCT was 268.2 ± 63.39 μm in the affected eye. Choroidal thickening (366 μm) and pachyvessels were observed in 1 patient. The mean central retinal thickness was 266.4 ± 35.73 μm. During the follow-up, one patient developed CNV as a complication, which responded to intravitreal ranibizumab treatment. Based on the OCT, 1 patient had conforming and 4 non-conforming type of FCE. We noticed a change of conformity in all patients, while in 4 patients we detected a change from non-conforming to conforming type. Among these, 3 experienced spontaneous changes, and 1 change was induced by ranibizumab treatment for CNV. In the patient with 2 non-conforming FCEs in 1 eye, a mild progression of hyporeflective space was detected in both FCEs during initial 3 years. However, in fourth year, the upper FCE spontaneously transitioned to a conforming type. Interestingly, the lower FCE maintained the same conformity throughout the follow-up period. In case of conforming FCE a hyporeflective space appeared twice between neuroretina and RPE with spontaneous regression.

**Table 1:** Characteristics of patients with FCE

No	Age	Vision complains	Coexisting maculopathy	OCT type	CCT (µm)	CRT (µm)	Refractive error (D)	Change of conformation on OCT	Fellow eye abnormality
1	63	no	no	non-conforming	258	264	+ 4.0	yes	no
2	56	no	pachychoroid	non-conforming	366	276	+ 3.0	yes	pachychoroid pigment epitelopathy
3	56	yes	CSCR	non-conforming	277	320	-5.0	yes	no
4	37	yes	choroid scar	non-conforming	249	224	0	yes	no
5	43	no	CNV	conforming	191	248	-4.0	yes	no

CCT- central choroidal thickness

CNV- choroidal neovascularization

CRT- central retinal thickness

CSCR- central serous chorioretinopathy

OCT- optical coherence tomography

### Case 1 - two asymptomatic FCEs in one eye

**Fig. 1:**
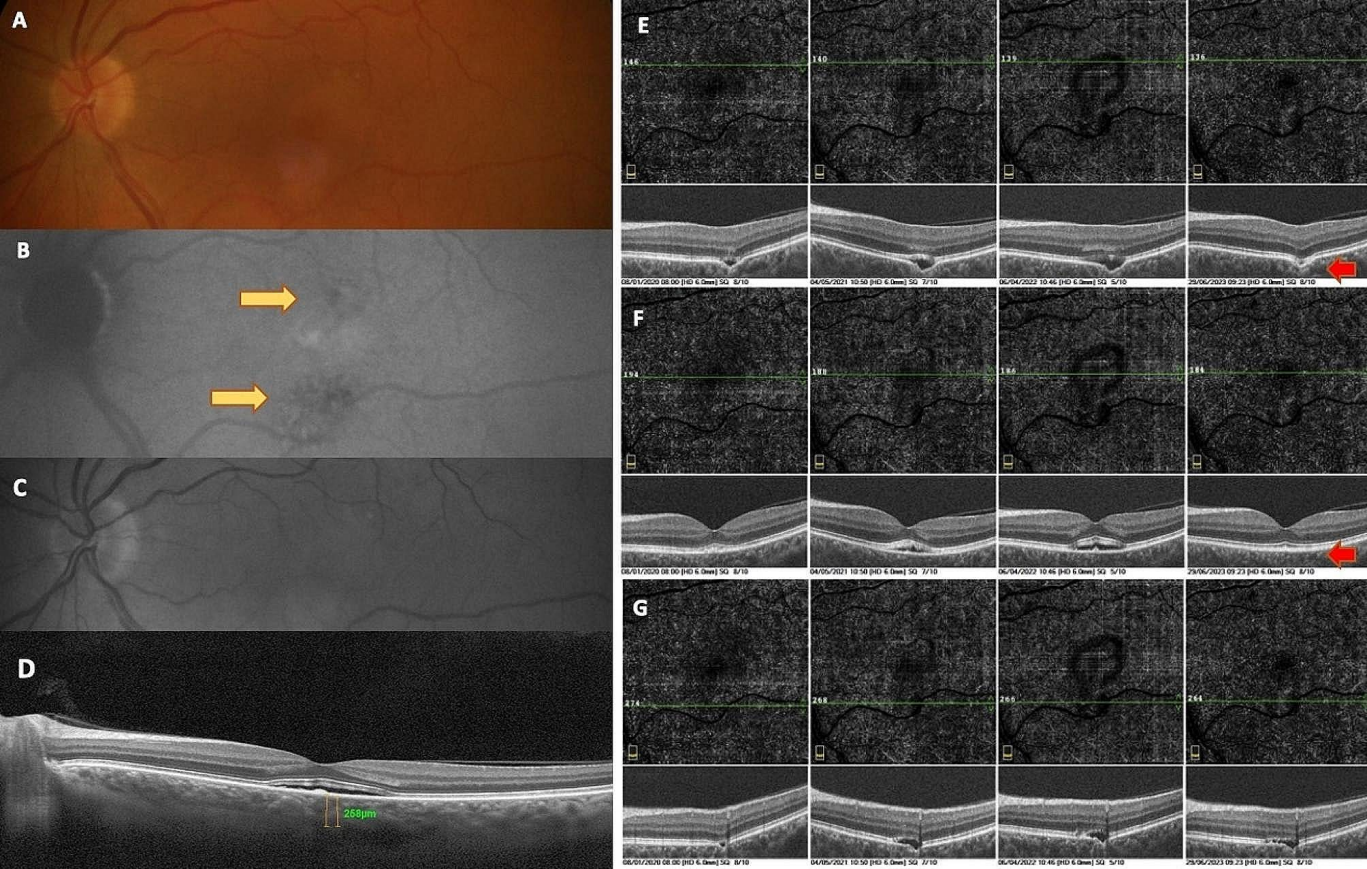
Case 1 - Multimodal imaging of patient with 2 FCE in 1 eye. The fundus photo (**A**) and FAF (**B**) illustrate irregularities of retinal pigment epithelium (RPE) above and below the foveola (yellow arrows). Red free image (**C**) shows no visible changes. EDI-OCT (**D**) reveals normal choroidal subfoveolar thickness and neuroretinal detachment at the centre. Over a 4-year OCT angiography follow-up, scans through the superior FCE (**E**), foveola (**F**) and inferior FCE (**G**) demonstrate mild progression of hyporeflective space in both FCEs during the first 3 years. In year 4, a spontaneous change to conforming FCE occurred in the upper FCE (red arrows)

In this case, a 63-year-old hyperopic (+ 4D) asymptomatic woman presented with bilateral BCVA 20/20. On the fundus photo and FAF, the RPE irregularities above and below the foveola were identified (Fig. 1). Choroidal thickness was found to be within the normal range. OCT revealed the presence of 2 FCEs– one situated above and one below the foveola, accompanied by a small neuroretinal detachment. Both excavations were initially non-conforming. OCT angiography displayed no CNV, so we opted to monitor only. Figure 1 also includes a 4-year OCT angiography follow-up, revealing a mild progression of hyporeflective space in both FCEs over the first 3 years, with no signs of CNV. Interestingly, in the fourth year, the upper FCE spontaneously converted to conforming type.

### Case 2 - pachychoroid FCE

**Fig. 2:**
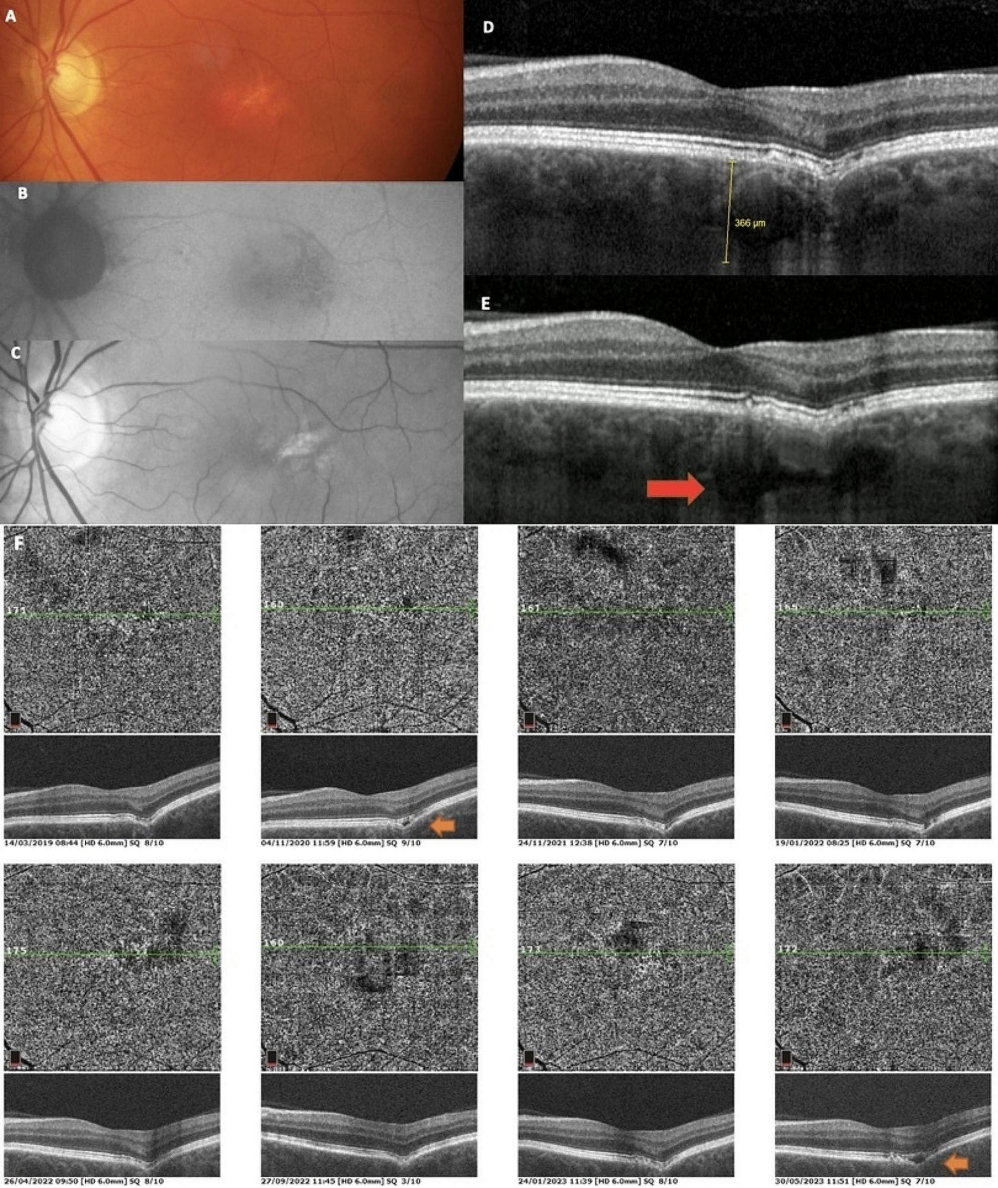
Case 2 - Multimodal imaging of patient with pachychoroid FCE. On fundus photo (**A**), FAF (**B**) and red free image (**C**), discrete RPE changes are observed. Subfoveolar choroidal thickness on EDI-OCT (**D**) is 366 μm and choroidal vessels with enlarged calibre are visible. Spectral OCT (**E**) shows conforming FCE with pachyvessels (red arrow). A 5-years OCT angiography (**F**) follow-up reveals a hyporeflective space appearing twice between neuroretina and RPE (orange arrows)

In case of a 56-year-old hyperopic (+ 3D) woman without visual complaints and with bilateral BCVA 20/20, discrete RPE changes were evident in the left eye on fundus photography, FAF and red free images (Fig. 2). Spectral OCT displayed conforming FCE. The subfoveolar choroidal thickness on EDI-OCT was 366 μm and pachyvessels were visible. This patient was incidentally diagnosed with pachychoroid FCE in left eye. Over the 5-year follow-up period, no progression of FCE was observed, and the visual acuity remained stable. However, OCT angiography revealed a spontaneous change to a non-conforming type, with the appearance of a hyporeflective space occurring twice between neuroretina and RPE during follow-up. The right eye macular OCT revealed the pachychoroid pigment epitelopathy.

### Case 3 - non-conforming FCE with CSCR, spontaneously changed to conforming FCE

**Fig. 3:**
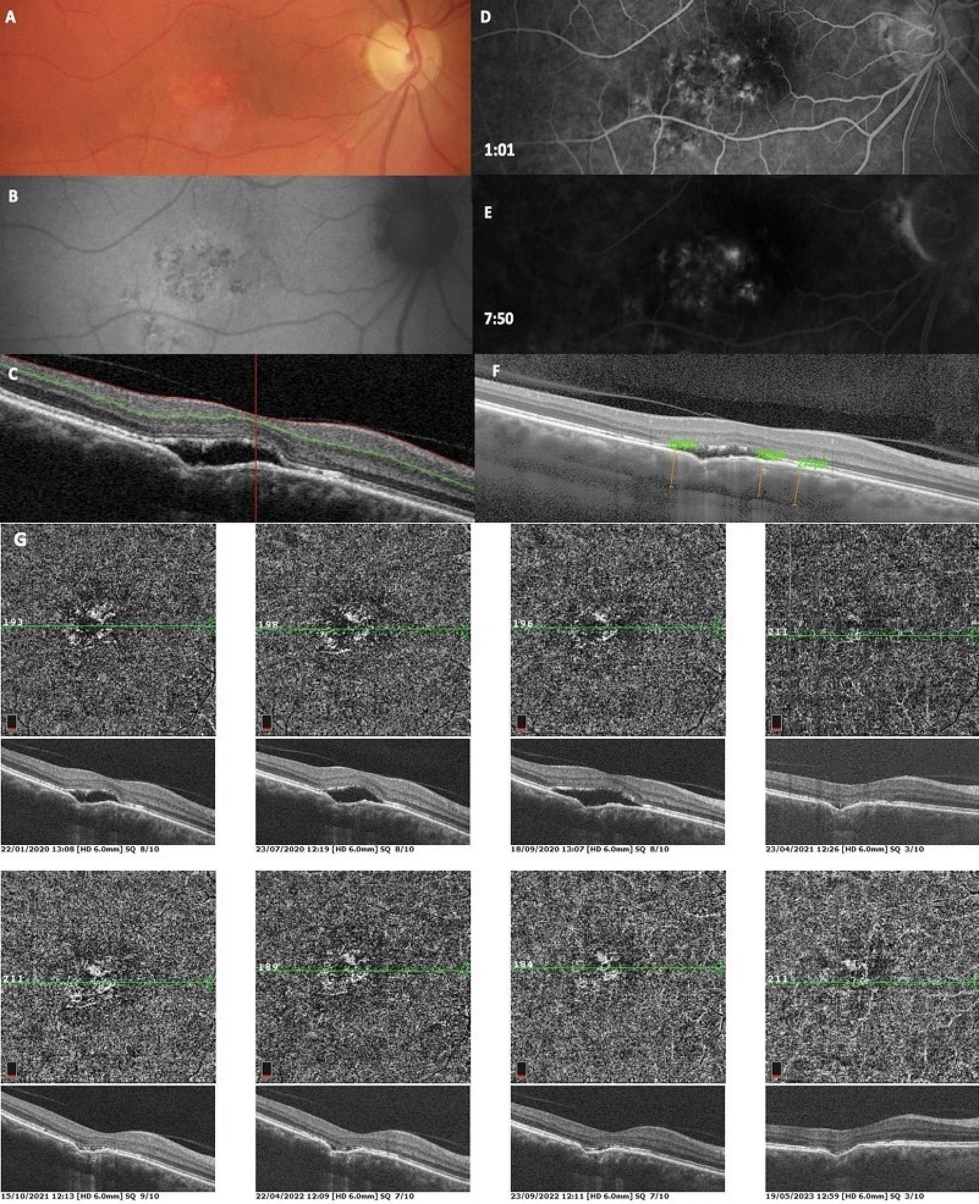
Case 3 - Multimodal imaging demonstrating non-conforming FCE with central serous chorioretinopathy (CSCR). Fundus photo (**A**) and FAF (**B**) display discrete pigment changes in the central area. Spectral OCT (**C**) shows central non-conforming FCE with hyporeflective space, with normal choroidal thickness on EDI-OCT (**F**). In the initial phase of FFA (**D**), pigmentepitelopathy and RPE irregularities are visible, and in late phase (**E**), the image shows leakage at the edge of FCE without clear detection of CNV. A 4-year follow-up on OCT angiography (**G**) records a spontaneous change from non-conforming to conforming FCE in choriocapillaris layer

A 56-year-old myopic with a refractive error of -5.0 D presented with complaint of decreased vision in the right eye persisting for 1 year, with BCVA 20/50. On the fundus photo and FAF a discrete pigmentary changes in central area were detected (Fig. 3). Spectral OCT showed a typical central non-conforming FCE with hyporeflective space. Normal subfoveolar choroidal thickness was demonstrated by EDI-OCT. Initial FFA revealed pigment epitelopathy and RPE irregularities, with late phase presentation indicating a leakage point at the edge of FCE without CNV, suggestive of CSCR. The patient was monitored without any treatment. No chorioretinal abnormalities were found in the fellow eye. Figure 3illustrates a spontaneous change from non-conforming to conforming FCE type during 4-year follow-up on OCT angiography.

### Case 4 - FCE in choroidal scar

**Fig. 4:**
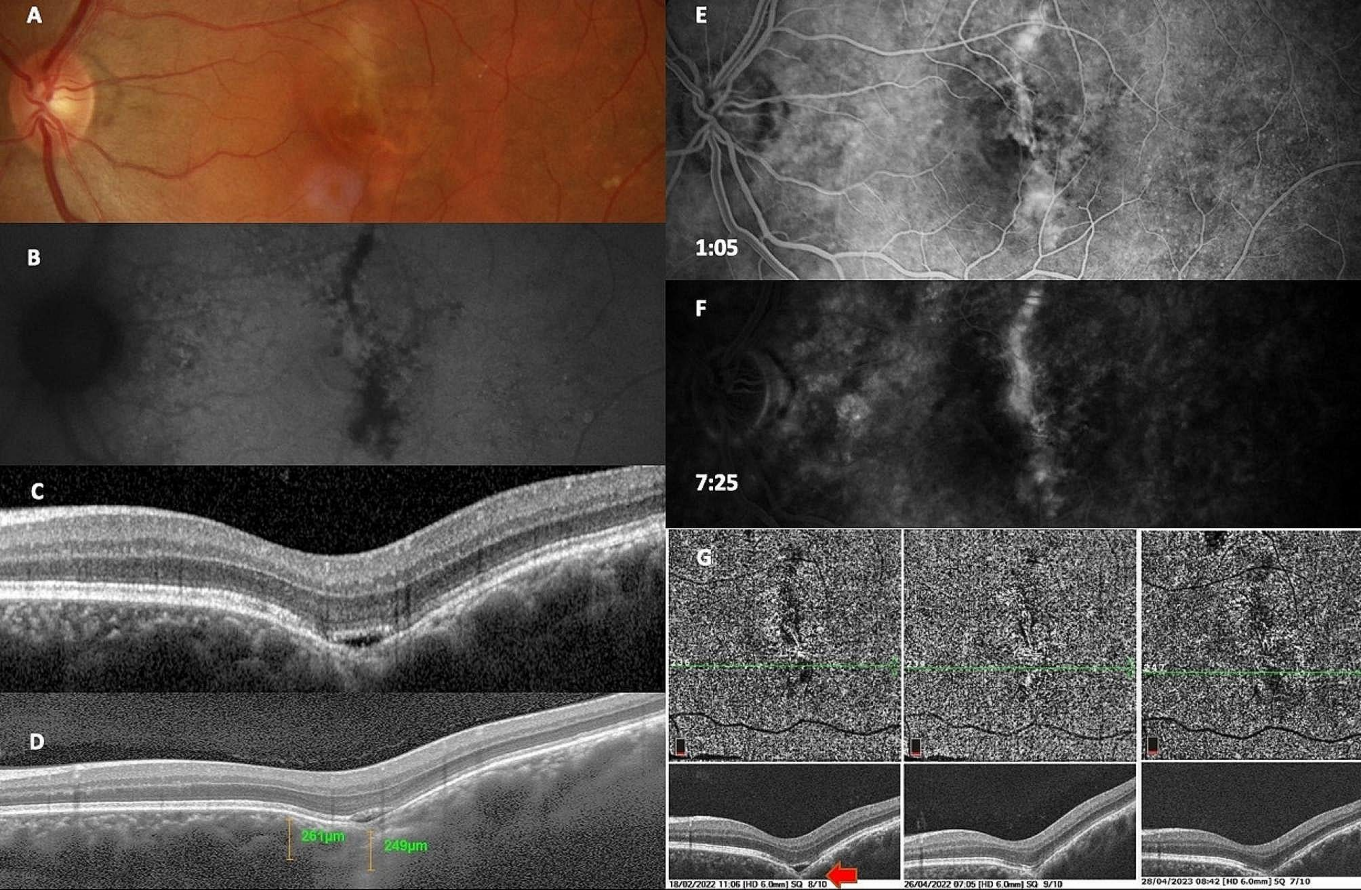
Case 4 - Multimodal imaging of patient with FCE in choroidal scar. Choroidal scarring and RPE atrophy are visible by fundus photo (**A**) and FAF (**B**). Spectral OCT (**C**) shows non-conforming FCE, ellipsoid zone and RPE atrophy. Subfoveolar choroidal thickness measured by EDI-OCT (**D**) is normal. FFA (**E**, **F**) displays fluorescein staining at the choroid scar without leakage. OCT angiography follow-up (**G**) records a change to conforming shape of FCE (red arrow)

A 37-year-old emmetropic man was diagnosed with an old choroidal scar of unknown origin in the left eye, with BCVA 20/100. No history of trauma, inflammation, or surgery was reported for the eye. Choroidal scarring and RPE atrophy were detected by fundus examination and FAF (Fig. 4). Spectral OCT of the left eye revealed non-conforming FCE, along with atrophy of ellipsoid zone and RPE. EDI-OCT did not detect pachyvessels, and the subfoveolar choroidal thickness measured 249 μm. On FFA fluorescein staining was detected at the site of the scar without any signs of leakage. CNV was not detected by OCT angiography and FFA. During the OCT angiography follow-up, a disappearance of the hyporeflective space was noted with conversion to conforming FCE type. The right eye exhibited no chorioretinal pathology with BCVA 20/20.

### Case 5 - FCE complicated by CNV

**Fig. 5:**
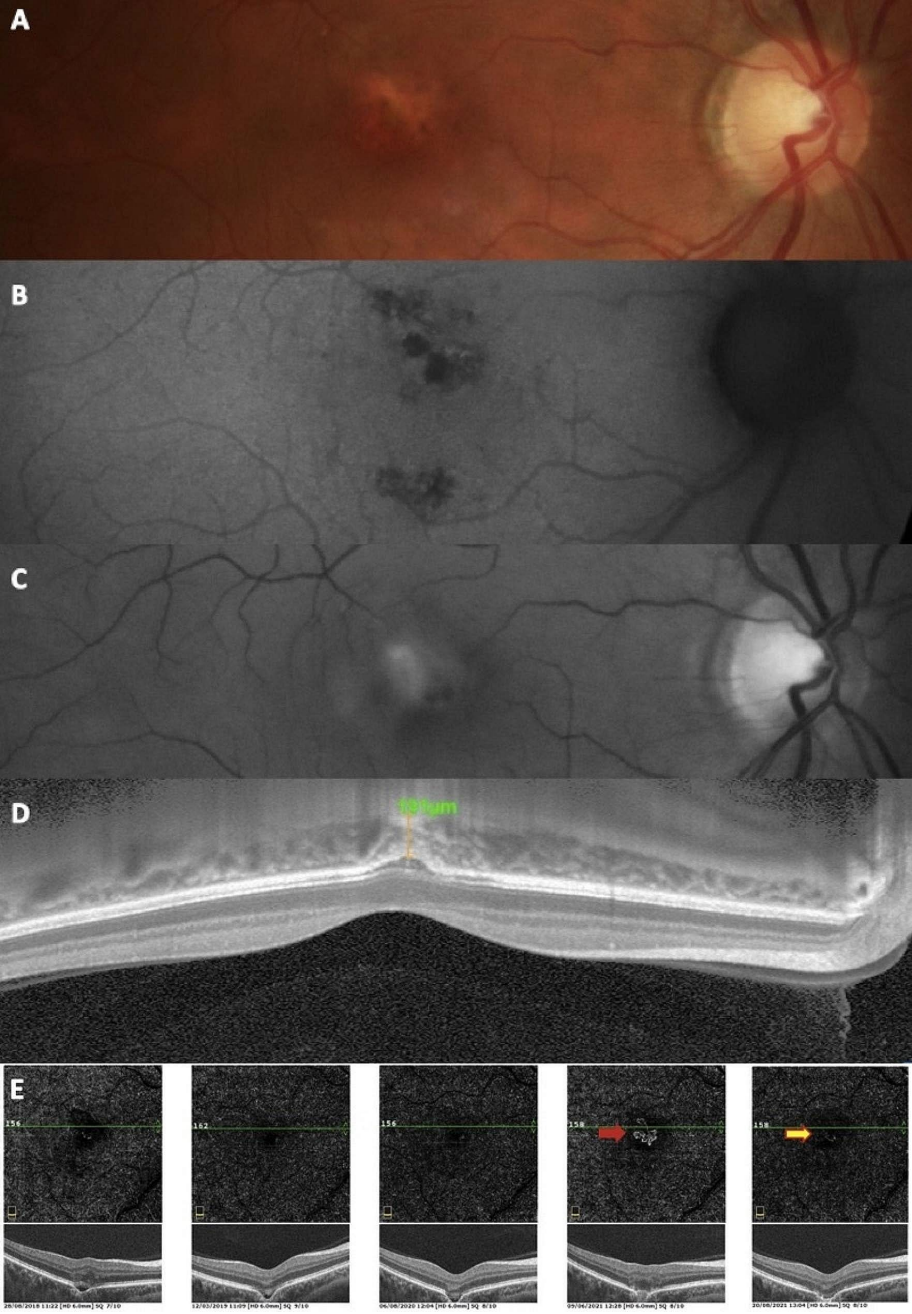
Case 5 - Multimodal imaging of a patient with FCE complicated by choroidal neovascularization (CNV). Fundus photo (**A**) demonstrates a small yellow lesion and pigment disruption in the foveola. On FAF (**B**), hypofluorescent areas are visible at the site of the disrupted RPE. No abnormalities are seen on the red free image (**C**). Subfoveolar choroidal thickness on EDI-OCT (**D**) is normal. A 4-year OCT angiography follow-up (**E**) shows a change to conforming FCE, CNV (red arrow) at the FCE, and finding 1 months after intravitreal ranibizumab (orange arrow)

A 43-year-old male patient with moderate myopia (-4.0 D) underwent a routine OCT examination in 2018, reporting no complaints, and with negative eye history. BCVA in both eyes was 20/20. On the fundus of the right eye, a small yellow lesion and pigment disruption in the foveola were detected (Fig. 5). Hypofluorescent areas at site of the disrupted RPE were revealed on FAF, while no abnormalities were observed on red free image. The thickness of the subfoveolar choroid measured 191 μm, remaining withing the normal range. The patient was regularly followed-up in 3-month intervals with non-conforming FCE. Figure 5illustrates a 4-year follow-up on OCT angiography from 2018 to 2021. In June 2021, CNV at the FCE site was detected and the patient was treated with 2 doses of ranibizumab, showing response. However, patient did not return for subsequent visits and was lost in follow-up. After ranibizumab treatment, the hyporeflective space disappeared. Examinations of the left revealed no chorioretinal abnormality or FCE.

## Discussion

The average age at the diagnosis of FCE is typically reported to be in the range of 40–50 years [3–5]. In our cohort, the mean age was 51 ± 10.65 years, slightly surpassing the commonly observed age bracket. Notably, Park and Oh identified FCE only in 3 out of 1697 adolescents (0.18%), with a mean age of 15.1 ± 11.2 years, emphasizing the rarity of FCE in younger patients and suggesting it may predominantly be an acquired condition [13]. Myopia is recognized as a risk factor for FCE, with approximately 58% of patients in studies reporting myopia [3–5], while in our group, 40% of patients were myopic.

Based on the OCT, we categorized FCE into 2 types - conforming and non-conforming type [3]. It remains uncertain whether these morphological types remain unchanged or change with time. Margolis et al. proposed that the non-conforming type is a more advanced stage of the conforming type, where the elasticity of the outer neuroretinal layers decreases, leading to the breakdown of the RPE and separation of the photoreceptors from the RPE [2]. The hyporeflective space in the non-conforming FCE represents serous retinal detachment due to improper RPE pumping action at the FCE [23, 24].

There are limited reports regarding the long-term course of eyes with FCE. While previous studies reported relatively stationary FCE lesions [3–5, 12, 25–27], our study introduces a novel perspective by revealing a dynamic nature in conformity for all examined patients. Obata et al. first demonstrated the relatively stationary nature of FCE through the examination of 21 eyes over an average period of 37 months. Their findings suggested minimal alterations in the appearance of FCE, with only 1 patient developed CSCR and 1 CNV [25]. Additionally, Park and Oh proposed that an increase in concavity size on OCT might result from focal scar contraction over time [13]. In contrast to these prior reports, our study demonstrates a change of conformity in all presented patients. While Margolis hinted at the potential for a transition from conforming to non-conforming type [3], there has been no explicit report of a reverse transition from non-conforming to conforming FCE. In out cohort, 4 patients with initially non-conforming FCE experienced a change to conforming type, with 3 cases occurring spontaneously and1 induced after ranibizumab. Conversely, in case of conforming FCE a change to non-conforming type happened twice with subsequent spontaneous regression. These observations provide evidence supporting the dynamic and reversible nature of FCE morphology on OCT.

FCE is a part of pachychoroid spectrum diseases and has been associated with condition such as CSCR or polypoidal choroidal vasculopathy (PCV). The formation of FCE in pachychoroid diseases is believed to be chronic venostasis, increased venous pressure and hyperpermeability of the pachychoroid vessels. This leads to increased exudation of pro-inflammatory cytokines into the choroidal stroma, resulting in degeneration, atrophy of the stroma and formation of FCE [15, 16]. The reported incidence of FCE in patients with CSCR is 1–8%, and 6% is in patients with PCV [12, 26, 27].

We have found that at the time of diagnosis, the most important examinations are OCT, OCT angiography and EDI-OCT. Fundus photos and red free imaging in patients with FCE were often nonspecific, while FAF images displayed unspecific irregular hypoautofluorescence. When diagnosing FCE, the choroidal thickness measurement by EDI-OCT has its own specifics, being thinnest at the base of the FCE, so we measure the subfoveolar choroid and around FCE. FFA findings of FCE were reported to be unremarkable in various studies, showing varying degrees of hyper or hypofluorescence [1, 3, 6, 28]. During follow-up, we predominantly relied on OCT angiography, a non-invasive method reflecting all retinal layers, able to detect CNV, and imaging the choroid thickness. CNV in FCE is believed to arise as a result of choroidal ischemia in the area of this anatomical anomaly [5, 6, 29]. One OCT sign typical for FCE, a hyperreflective tissue beneath the lesion, suggesting scarring of choroidal connective tissue resulting from previous inflammation or ischemia, was not observed in our patients, as described by Ellabban [26]. In light of our observations confirmed on OCT angiography, we recommend monitoring FCE patients every 3–6 months and start treatment when CNV is conclusively confirmed.

The strength of our study is a long-term follow-up, consistent data collection and high-quality imaging. However, the study is limited by its small sample size and retrospective nature.

## Conclusion

This study confirms the occurrence of asymptomatic changes in the shape of FCE, transitioning between conforming to non-conforming FCE and vice versa. We interpret these subtle alteration in hyporeflective space as non-pathologic and clinically insignificant. For cases of asymptomatic FCE without associated choroidal neovascular changes, we recommend observation with no treatment. Additionally, we consider the use of OCT angiography as the optimal method to monitor patients with FCE and detect potential CNV.

## Data Availability

All data analyzed during this study are included in this article and also available from the corresponding author on reasonable request.
